# Effects of Very Low-Calorie Diet versus Roux-en-Y Gastric Bypass Surgery on Body Composition in Patients with Obesity

**DOI:** 10.3390/nu16152407

**Published:** 2024-07-25

**Authors:** Chanawit Saiyalam, Prapimporn Chattranukulchai Shantavasinkul, Supphamat Chirnaksorn, Ploysyne Rattanakaemakorn, Naphat Taonam, Vorachat Rodphech, Supanee Putadechakum, Sasivimol Rattanasiri, Jintana Sirivarasai, Boonsong Ongphiphadhanakul, Preeda Sumritpradit

**Affiliations:** 1 Doctor of Philosophy Program in Nutrition, Faculty of Medicine Ramathibodi Hospital and Institute of Nutrition, Mahidol University, Bangkok 10400, Thailand; chanawit.sai@student.mahidol.ac.th; 2 Division of Nutrition and Biochemical Medicine, Department of Medicine, Faculty of Medicine Ramathibodi Hospital, Mahidol University, Bangkok 10400, Thailand; nutrinaphatta@gmail.com (N.T.); pvorachat.rod@gmail.com (V.R.); supanee.put@mahidol.ac.th (S.P.); 3 Graduate Program in Nutrition, Faculty of Medicine Ramathibodi Hospital, Mahidol University, Bangkok 10400, Thailand; jintana.sir@mahidol.ac.th; 4 Division of Gastroenterology, Department of Medicine, Faculty of Medicine Ramathibodi Hospital, Mahidol University, Bangkok 10400, Thailand; supphamat.chi@gmail.com; 5 Division of Dermatology, Department of Medicine, Faculty of Medicine Ramathibodi Hospital, Mahidol University, Bangkok 10400, Thailand; ploysyne@gmail.com; 6 Department of Clinical Epidemiology and Biostatistics, Faculty of Medicine Ramathibodi Hospital, Mahidol University, Bangkok 10400, Thailand; sasivimol.rat@mahidol.ac.th; 7 Division of Endocrine and Metabolism, Department of Medicine, Faculty of Medicine Ramathibodi Hospital, Mahidol University, Bangkok 10400, Thailand; boonsong.ong@mahidol.ac.th; 8 Department of Surgery, Faculty of Medicine Ramathibodi Hospital, Mahidol University, Bangkok 10400, Thailand; psumritpradit@gmail.com

**Keywords:** very low-calorie diet, Roux-en-Y gastric bypass, bariatric surgery, metabolic and bariatric surgery, body composition, diabetes, type 2 diabetes, diabetes remission

## Abstract

Roux-en-Y gastric bypass (RYGB) is the most effective treatment for severe obesity. A very low-calorie diet (VLCD) is another effective dietary intervention to treat obesity. This study evaluated the effect of a VLCD versus RYGB on weight reduction, changes in body composition and the resolution of comorbidities during a 12-week period. Individuals with obesity at the obesity clinic, Ramathibodi Hospital, Mahidol University, Thailand with a body mass index (BMI) ≥ 37.5 kg/m^2^ or ≥32.5 kg/m^2^ with obesity-related complications were recruited. Treatment options, either RYGB or VLCD, were assigned depending on patients’ preferences and physicians’ judgment. The analysis included 16 participants in the RYGB group and 15 participants in the VLCD group. Baseline characteristics were similar between groups; nevertheless, the participants in the VLCD group were significantly younger than those in the RYGB group. The number of patients with type 2 diabetes (T2D) was slightly higher in the RYGB group (43.8% vs. 33.3%, *p* = 0.552). Additionally, patients in the RYGB group had a longer duration of T2D and were treated with anti-diabetic agents, while VLCD patients received only lifestyle modifications. At 12 weeks, total and percentage weight loss in the RYGB and VLCD groups, respectively, were as follows: −17.6 ± 6.0 kg vs. −15.6 ± 5.1 kg (*p* = 0.335) and −16.2% ± 4.3% vs. −14.1% ± 3.6% (*p* = 0.147). Changes in biochemical data and the resolution of comorbidities were similar between the groups at 12 weeks. A 12-week VLCD resulted in similar weight loss and metabolic improvement compared with RYGB. Large-scale studies with long follow-up periods are needed to elucidate whether VLCD is a viable alternative treatment to bariatric surgery.

## 1. Introduction

Obesity is a chronic and progressive disease [1] resulting from excessive fat accumulation and adipose tissue dysfunction [2]. It is a major public health problem that affected approximately 890 million people globally in 2022 [3]. The prevalence of obesity in Thailand, as defined by a body mass index (BMI) of at least 25 kg/m^2^, was approximately 46.4% in 2020 [4]. Metabolic and bariatric surgery (MBS), in particular Roux-en-Y gastric bypass (RYGB), is the most effective treatment for severe obesity, and it results in significant and sustained weight loss [5,6]. In addition, RYGB can reverse obesity-related complications and, in particular, result in the remission of type 2 diabetes (T2D) [7,8]. However, MBS has a high cost and is restricted to people with severe obesity. Moreover, some patients may have contraindications to the operation and other patients may refuse surgical intervention.

A very low-calorie diet (VLCD) is one of the most effective dietary interventions to treat people with severe obesity. It can induce rapid weight loss ranging from 8.4 to 17 kg within 8–12 weeks [9,10,11]. Thus, it is suitable for individuals with severe obesity who have already attempted to lose weight without achieving weight loss targets or for individuals with weight regain. VLCD typically restricts calorie intake to less than 800 kcal per day to induce rapid weight loss and ameliorate obesity comorbidities [12]. In patients undergoing MBS, 2 weeks of preoperative weight loss using VLCD can significantly reduce weight, liver volume, operation time and surgical difficulty [13,14,15,16].

A VLCD can be prescribed as a total meal replacement or a food-based diet. However, meal replacements, typically formulated as prepackaged shakes or bars, are commonly used since they help promote weight loss by controlling portions and eliminating food choices. In the past, VLCD was commonly used as an in-hospital, weight loss program. Recently, many studies have confirmed the efficacy and safety of an intensive weight loss program using VLCD as a total meal replacement in an outpatient setting [16,17,18,19]. 

To date, only a few studies have compared the efficacy and safety of VLCDs and RYGB, particularly in an Asian population. Therefore, the present study compared the effect of a VLCD versus RYGB on weight loss, body composition, metabolic changes and the resolution of comorbidities during a 12-week study period.

## 2. Materials and Methods

### 2.1. Participants

This study was conducted at the obesity clinic, Ramathibodi Hospital, Mahidol University, Bangkok, Thailand. We enrolled individuals 15–65 years of age with obesity and a BMI ≥ 37.5 kg/m^2^ or ≥32.5 kg/m^2^ with obesity-related complications such as T2D, hypertension, dyslipidemia, sleep apnea or non-alcoholic fatty liver disease. Exclusion criteria were type 1 diabetes, weight loss ≥5% in the previous 3 months, chronic kidney disease (estimated glomerular filtration rate < 30 mL/min/1.73 m^2^), anti-obesity medications, prebiotics and probiotics, uncontrolled psychiatric diseases, substance abuse, previous bariatric surgery, pregnancy or lactation, and allergies to any constituent of the meal replacement products. 

The study was approved by the Human Research Ethics Committee, Faculty of Medicine Ramathibodi Hospital, Mahidol University and was registered on ClinicalTrial.gov (NCT05459675). The study protocol was explained in detail to the participants, and they were allowed to ask questions. All questions were answered to the patient’s satisfaction. All participants voluntarily provided written informed consent and the study was performed in accordance with the principles of the Declaration of Helsinki.

### 2.2. Intervention

All participants in the present study were offered all treatment options for obesity, including lifestyle modification, anti-obesity medications and MBS. In this study, we included only individuals with obesity who were interested in VLCD or bariatric surgery. Treatment options, either RYGB or a 12-week VLCD, were assigned to each individual depending upon the patient’s preference and the physician’s judgment. The follow-up visits were at baseline, and at 4 and 12 weeks after RYGB or initiation of VLCD.

#### 2.2.1. Very Low-Calorie Diet Group

Participants in the VLCD group received a total meal replacement using a low-energy formula diet of three meals per day for 12 weeks (Nestle Boost Care^®^ (Kolnofingen, Switzerland) with Nestle Boost Bene Protein^®^ (WI, USA): 1 packet provided 247 kcal, protein 30 g, fat 7 g and carbohydrate 16 g). In addition to the formula diet, the participants were asked to consume at least two cups of non-starchy vegetables and one teaspoon of vegetable oil, two tablets of multivitamins and minerals, and drink at least 2–3 L of water per day. After 2–4 weeks, the physician determined whether the participants should receive additional dietary protein intake on physician discretion and patient’s preference. Food re-introduction was gradually increased from week 8 through to week 12. Nevertheless, all patients continued taking at least two meal replacements per day and the caloric intake was controlled to be under 900 kcal/day throughout the study period.

#### 2.2.2. Roux-en-Y Gastric Bypass Group

All participants underwent laparoscopic RYGB performed by one experienced surgeon in the following manner: a 30 mL proximal gastric bypass pouch was created and the jejunum was divided 50 cm distal to the ligament of Treitz. A Roux-en-Y gastrojejunostomy was created between the gastric pouch and the distal segment of the divided jejunum. Gastrointestinal continuity was restored by creating a stapled side-to-side jejunojejunostomy, resulting in a 150 cm alimentary limb and a 50 cm biliopancreatic limb. After surgery, a staged meal progression was prescribed according to the nutrition recommendation after bariatric surgery: a liquid diet, a pureed diet, a soft diet and a regular high-protein diet. The participants were also prescribed multivitamins and minerals, calcium, vitamin D, iron and vitamin B12 supplementation as part of the post-bariatric nutrition regimen [20,21,22].

### 2.3. Weight and Body Composition Measurements

Anthropometric parameters, including weight, height and waist circumference, were measured using standard techniques at each follow-up visit. Body composition was determined after at least 8 h of fasting using multifrequency bioelectrical impedance analysis with eight-point tactile electrodes (InBody 770; Biospace, Seoul, Republic of Korea). Body fat percentages (%BF) and skeletal muscle mass percentages (%SMM) were calculated by (body fat mass/weight) × 100 and (SMM/weight) × 100, respectively. This study presents the data on weight loss as total weight loss (kg) and percentage weight loss (%WL). BMI was calculated by weight (kg)/(height in meters)^2^, and %WL was calculated from baseline weight.

### 2.4. Dietary Assessment and Blood Chemistry Measurement

Dietary intakes were assessed using a 24 h dietary recall at each follow-up visit. Caloric intake and macronutrient composition were assessed by an experienced registered dietitian. Venous blood samples for biochemical tests were measured at baseline and at 12 weeks. Fasting plasma glucose was measured by the hexokinase method using an automated machine. Hemoglobin (Hb) A1c was measured by immunoassay (turbidimetric inhibition immunoassay (TINIA), Tina-quant^®^, Roche, Basel, Switzerland). Serum biochemical testing as well as fasting plasma lipid profiles were measured using the Abbott Alinity c system (Abbott Laboratories, Abbott Park, IL, USA).

### 2.5. Diabetes Remission

Currently, T2D remission is defined as an HbA1c < 6.5% measured at least 3 months after surgery or 6 months after lifestyle intervention and cessation of any glucose-lowering agents [23]. In our study, we defined patients as being in T2D remission if they had an HbA1c < 6.5% and had no glucose-lowering agents at 12 weeks after intervention.

### 2.6. Statistical Analysis

Statistical analyses were performed using the STATA software package, version 18.0 (Stata Corp, College Station, TX, USA). Data are presented as mean ± standard deviation (SD) or median and interquartile range for continuous variables and frequency (%) for categorical variables. The continuous variables were compared using an independent sample *t*-test for normal distribution; otherwise, quantile regression was applied to compare the median between two independent groups. The test results of categorical variables were evaluated by a χ^2^ or Fisher’s exact test as appropriate. Multilevel mixed-effects linear regression was used to assess the difference in continuous outcomes at 4 and 12 weeks. The interaction between the time and group was included. The results were deemed statistically significant at *p* < 0.05.

## 3. Results

### 3.1. Characterization of the Participants

**Figure 1:**
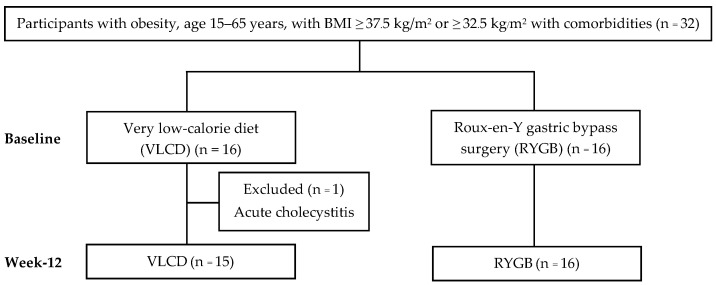
Participant flow diagram.

A total of 32 participants were recruited for the study. However, we excluded one subject in the VLCD group from the analysis since they developed acute cholecystitis and underwent laparoscopic cholecystectomy at 3 weeks after initiation of VLCD. Thus, 15 participants in the VLCD group and 16 participants in the RYGB group were included in the analysis (Figure 1). Baseline characteristics and biochemical data were similar between the groups (Table 1 and Table 2). For all participants, mean ± SD age was 35.2 ± 8.2 years, and the majority of participants were female (71%). Body weight and BMI were 110.3 ± 24.1 kg and 40.5 ± 7.4 kg/m^2^, respectively. The participants in the RYGB group were significantly older than those in the VLCD group. Moreover, the number of patients with T2D was slightly higher in the RYGB group (RYGB 7/16; 43.8% vs. VLCD 5/15; 33.3%, *p* = 0.552) (Table 1). 

### 3.2. Study Endpoints

**Figure 2:**
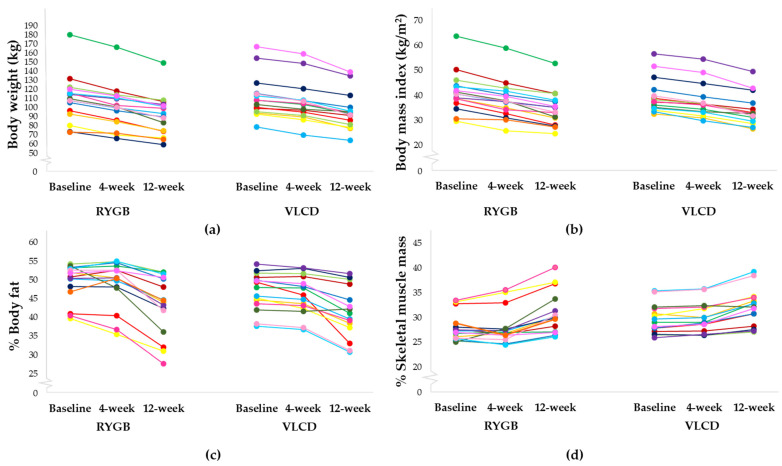
Individual changes in body weight, body mass index and body composition at baseline, 4 weeks and 12 weeks for the RYGB group and VLCD group: (**a**) body weight change, (**b**) BMI change, (**c**) % body fat change, (**d**) % skeletal muscle mass change.

Total weight loss and total BMI loss at 12 weeks for the RYGB and VLCD groups, respectively, were as follows: −17.6 ± 6.0 kg vs. −15.6 ± 5.1 kg (*p* = 0.335) and −6.7 ± 2.2 kg/m^2^ vs. −5.6 ± 1.6 kg/m^2^ (*p* = 0.114). Interestingly, participants in the RYGB group had significantly higher %WL at 4 weeks (RYGB; −7.9 ± 3.1% vs. VLCD; −5.3 ± 2.0%, *p* = 0.009) but not at 12 weeks (RYGB; −16.2 ± 4.3% vs. VLCD; −14.1 ± 3.6%, *p* = 0.147). All participants in both groups had achieved at least 5% weight loss at 12 weeks. A total of 93.8% of participants in the RYGB group and 86.7% in the VLCD group had achieved at least 10% weight loss at 12 weeks. Figure 2 shows the changes in body composition in each individual according to the RYGB and VLCD groups at baseline, 4 weeks and 12 weeks.

Body weight, BMI, BF and SMM were similar between groups at study entry. At 4-week and 12-week time points, the VLCD group had significantly higher weight compared with the RYGB group. By contrast, BMI and BF were not different between groups throughout the study period. Interestingly, the VLCD group had significantly lower %BF and significantly higher SMM and %SMM at 4 weeks and 12 weeks post intervention. All these findings corresponded to differences in dietary intakes between the groups (Table 3), even though the caloric intakes were comparable between groups at baseline. After RYGB, total calories and all macronutrient compositions were significantly reduced at 4 weeks and 12 weeks (average caloric intake 300–700 kcal/day with protein intake 20–44 g/day). For the VLCD group, the caloric intake was maintained at approximately 800–900 kcal/day, with total protein 96–120 g/day. The caloric and protein intakes of the VLCD group were significantly higher than those of the RYGB group throughout the study period (Table 3).

There were 7/16 (43.8%) patients in the RYGB group and 5/15 (33.3%) patients in the VLCD group who had pre-existing T2D. At 12 weeks, T2D remission was achieved by 4/7 (57%) in the RYGB group and 4/5 (80%) in the VLCD group. For patients with T2D, baseline fasting plasma glucose and HbA1c were slightly higher in the VLCD group (Table 2). In addition, all the T2D patients in the VLCD group had been recently diagnosed with T2D and all had been treated by lifestyle modification. By contrast, most of the T2D patients in the RYGB group were treated with oral hypoglycemic agents, glucagon-like peptide-1 (GLP-1) receptor agonists and/or insulin (Table 1). For subjects with and without T2D, both fasting plasma glucose and HbA1c were significantly reduced after RYGB and VLCD. However, the degree of reduction was more obvious in T2D patients (Table 4).

For patients with pre-existing hypertension at study entry, a total of five out of nine (55.6%) in the RYGB group and three out of four (75%) in the VLCD group had hypertension resolution. For both groups, all patients had improvements in blood pressure control, which was defined as a reduction in anti-hypertensive medications or significantly lowered blood pressure levels compared with baseline.

After all the participants had completed the 12-week study period, we routinely followed them at our obesity clinic. The follow-up rates of the participants in the RYGB group and VLCD group were 16/16 (100%) and 13/15 (86.7%) at 6 months and 14/16 (87.5%) and 9/15 (60%) at 12 months, respectively. Weight regains were more common in the VLCD group at 6 months and 12 months. For the RYGB group, all participants were able to maintain their weight loss at 12 months, regardless of whether they continued attending the clinic or were lost to follow-up. In contrast, for the VLCD group, all six patients who were lost to follow-up at 12 months experienced weight gain compared to their 3-month visit. Nevertheless, only 22.2% of the patients who continued attending the clinic experienced significant weight gain at 12 months. A total of 3/15 (20%) participants in the VLCD group had significant weight regain and required additional weight loss intervention at 12 months. One patient was interested in MBS and two participants required anti-obesity medications including GLP-1 receptor agonists.

### 3.3. Adverse Events

In the VLCD group, one patient developed acute cholecystitis and underwent laparoscopic cholecystectomy without any complications at 3 weeks after initiation of the VLCD. Therefore, we excluded this patient from the analysis. No other serious adverse events were observed in either group. There were no changes in serum creatinine or electrolytes during the study period. Adverse events in the RYGB group were abdominal discomfort (56.3%), nausea (43.8%), vomiting (25%), dumping syndrome (25%) and constipation (25%). Whereas adverse events in the VLCD group were fatigue (33.3%), sleepiness (33.3%), diarrhea (33.3%) and nausea (13.3%).

## 4. Discussion

This study demonstrated that a 12-week VLCD resulted in similar total weight loss, %WL, metabolic improvement and resolution of comorbidities compared with RYGB. The VLCD group had significantly lower %BF and significantly higher SMM and %SMM at 4-weeks and 12-weeks post intervention. Moreover, T2D remission was achieved in patients in both groups. Other cardiovascular risk factors including blood pressure and lipid profiles were also improved in both groups. However, more patients were lost to follow-up or experienced weight regain in the VLCD than the RYGB group at 6-months and 12-months post intervention.

In this study, the VLCD intervention was total diet replacement combined with non-starchy vegetables and 1 teaspoon of vegetable oil; it contained 800–900 kcal and 90 g/day of protein. The study diet was effective in terms of reducing weight and improving metabolic parameters, comparable to RYGB. In clinical practice, a VLCD can be prescribed as a nutritionally complete very low-calorie formula as total diet replacement or standard diet. Both diets provide similar results in weight loss outcomes and metabolic improvement. Nevertheless, a formula diet may be better in terms of feasibility since it helps patients to control portions, and the patients do not need to count caloric intake, choose a healthy high-protein diet or prepare meals. Average weight loss using VLCD for 12–20 weeks can induce rapid weight loss as well as a reduction in liver volume, visceral fat and total body fat, and improve obesity-related complications [24,25,26,27,28]. Several studies have demonstrated that VLCDs can produce remission of T2D [16,18,19] lasting up to 2 years [29].

The safety of short-term VLCDs, either using a nutritionally complete formula or a nutrient-rich diet, has been confirmed in several studies [16,17,18,19,29,30]. It does not cause protein-calorie malnutrition [31,32]. Nonetheless, VLCDs could induce loss of lean body mass, which may lower the metabolic rate and have detrimental effects on long-term weight loss outcomes. Therefore, a VLCD formula containing high levels of protein should be used to ensure that patients maintain their lean body mass during the rapid weight loss phase. In addition, hydration status and serum electrolytes, particularly potassium and magnesium, should be monitored. Multivitamins and minerals should be supplemented in all patients. Moreover, anti-diabetic medications, especially insulin, and anti-hypertensive medications can be reduced or even stopped shortly after the start of the VLCD. Self-monitoring blood glucose and home blood pressure monitoring should be suggested for patients with pre-existing T2D or hypertension who are currently taking anti-diabetic or anti-hypertensive medications.

MBS is the most effective treatment to induce significant weight loss and improve obesity-related complications in patients with severe obesity. RYGB is a commonly performed bariatric procedure worldwide and it significantly induces profound changes in appetite regulation, food preference, and pancreatic and gut hormone secretion following the operation. The decrease in appetite and caloric intake plays a crucial role in significant and sustained weight loss after RYGB. Additionally, enhanced postprandial GLP-1 and peptide YY are responsible for body weight reduction, improvement in glucose regulation and T2D remission [33,34,35]. A recent study revealed that RYGB resulted in divergent brain responses compared with VLCD-induced weight loss, which may explain weight regain after VLCD and sustained weight loss after RYGB [9].

Total caloric and macronutrient intakes decreased significantly during the first 1–3 months after RYGB, leading to rapid and substantial weight loss in the post-operative period [36]. This weight loss continues and reaches its maximum at 1–2 years after the operation [37,38]. Patients undergoing MBS should receive education from a registered dietitian on meal progression, emphasizing three small meals per day. A healthy diet high in protein, with a minimum of 60 g per day or up to 1.5 g per kilogram of ideal body weight per day, along with multivitamin and mineral supplementation, should be recommended [21]. Despite intensive dietary counseling, low protein intake is common [39] among patients undergoing metabolic and bariatric surgery (MBS), often due to decreased overall food intake, diet intolerance and aversions to protein-rich foods such as meat. This can lead to muscle loss and sarcopenia. Severe protein malnutrition, though rare, can result in significant morbidity. In our study, participants in the RYGB group experienced a marked reduction in both caloric and protein intakes throughout the study period (300–700 kcal with 20–44 g or protein per day). This is consistent with previous research, which reported average energy intakes of approximately 625 to 680 kcal and protein intakes of 29 to 30 g/day at 1 month, and 722 to 1047 kcal and 35 to 45 g/day at 3 months after MBS [36,39,40]. 

Protein intake significantly decreased immediately post surgery through the 1-month post-operative period, with patients typically gradually increasing their intake by 3–6 months. Our study also found that protein intake in the RYGB remained below the recommended minimum during the 12-week period. In contrast, the VLCD group maintained their caloric intake approximately 800–900 kcal with 96–120 g of protein mainly from total diet replacement. The difference in caloric and protein intake between group may be the responsible factor that the VLCD group had significantly lower %BF and significantly higher SMM and %SMM at 4 weeks and 12 weeks post intervention. To mitigate this, protein supplementation could play a crucial role in increasing intake and preventing muscle loss and protein malnutrition during the early recovery phase after RYGB. 

Other factors potentially influencing differences in weight loss outcomes and changes in body composition between groups include self-motivation, self-efficacy and health literacy. Previous studies [41,42,43,44,45] have suggested that these factors may impact weight loss outcomes following MBS and intensive lifestyle modifications. Given the non-randomized design of our study, we cannot rule out potential variations in self-motivation, self-efficacy and health literacy among participants in the RYGB and VLCD groups.

Our study demonstrates that a VLCD using total diet replacement is well tolerated and effective in inducing rapid and significant weight loss. It could be an alternative option for patients with severe obesity who are unable to undergo bariatric procedures. Otherwise, a VLCD could be used as an initial treatment for weight loss, with other strategies added in the weight loss maintenance phase. In our study, patients in the VLCD group who were followed in the clinic maintained their weight loss at 12 months. Once participants in the VLCD group gained more than 5% above their lowest weight, we had a rescue plan to facilitate weight loss maintenance. However, patients in the VLCD group who were lost to follow-up tended to regain significant amounts of weight. This is similar to the previous studies indicating that participants who attend clinics more regularly tend to achieve improved long-term weight loss outcomes following either MBS or intensive lifestyle interventions [46,47]. The findings from our study underscore the importance of implementing an active, structured follow-up program to enhance patient adherence and mitigate the risk of weight regain. Furthermore, it is essential to have an additional intervention, such as restarting VLCD or considering anti-obesity medications, for patients who experience weight regain after discontinuing a VLCD.

Diabetes remission was slightly more common in the VLCD group than the RYGB group (VLCD 4/5 (80%) vs. RYGB 4/7 (57%); *p* = 0.916), even though the difference was not significant because of the small number of patients who had T2D. The difference can be explained by the fact that all patients in the VLCD group had recently been diagnosed with T2D and all had been treated with only lifestyle modification. Furthermore, T2D duration was significantly longer in the RYGB group. Moreover, the patients with T2D in the RYGB group were treated with either oral hypoglycemic agents, GLP-1 receptor agonists and/or insulin. Our findings confirm those of previous studies, which have indicated that the predictors of T2D remission after either VLCD or MBS are a short duration of T2D, not currently taking insulin and a higher *C*-peptide level [48,49]. All these factors may indicate the preservation of beta-cell function in individuals recently diagnosed with T2D [50].

A significant improvement of glucose control was observed in patients with T2D as early as 7 days after the start of VLCD. This is primarily because of enhanced beta-cell function [51], decreased hepatic glucose production [52] and increased insulin sensitivity [53]. Moreover, by causing substantial weight loss, VLCD significantly reduces ectopic fat, in particular hepatic and pancreatic fat, and restores hepatic insulin sensitivity and beta-cell function [54]. The mechanisms underlying T2D remission after MBS could be explained by sudden caloric restriction, which is a mediator of early improvement in glucose control via a reduction in hepatic glucose production [55]. Additionally, structural changes in the gastrointestinal tract lead to an enhanced postprandial incretin response, particularly in GLP-1, which interacts with the brain to result in a reduction in appetite and food intake. Moreover, GLP-1 stimulates insulin secretion and suppresses glucagon, which leads to better glucose control.

Our study illustrated that RYGB and VLCD could induce T2D remission and treat other obesity-related complications. Nevertheless, RYGB typically results in substantial and durable weight loss, whereas a VLCD often leads to significant but short-term weight reduction, necessitating additional intervention to prevent weight regain. Therefore, we would like to propose that RYGB may be a suitable option for individuals with severe obesity who have made multiple unsuccessful attempts at weight loss, experienced weight regain or express willingness to undergo MBS and are committed to post-operative follow-up to mitigate surgical complications such as vitamin and mineral deficiencies. Conversely, a VLCD is a viable option for motivated patients with severe obesity who are not yet prepared for an invasive procedure, those with contraindications to surgery or individuals unable to afford the associated costs of the operation. Moreover, VLCD can be used as a preoperative weight loss approach prior to undergoing MBS.

Gall bladder disease including gall stones, acute cholecystitis or even gall stone pancreatitis has been reported to be a common complication after rapid weight loss either with lifestyle modification [56,57], weight loss medication (particularly GLP-1 receptor agonist) [58,59] or MBS [60,61]. Uncertainty surrounds the exact pathophysiology of gall stone development after rapid weight loss. Several recognized mechanisms contribute to gall stones, including an increase in bile composition and concentration [62,63], which results in the formation of cholesterol monohydrate crystals and gall bladder dysfunction [56,64,65].

The strength of our study was that we prospectively enrolled individuals with obesity who were potential bariatric candidates and directly compared the intensive medical intervention and MBS. Moreover, the patients’ compliance was very high during the 12-week follow-up period. We acknowledge potential limitations of our study. First, there was potential bias since we did not randomize the patients and we could not blind either investigators or participants. Nevertheless, obesity treatment is complex and health care providers should emphasize the importance of a personalized approach to address the unique needs of each patient. This is why we decided to let patients primarily select treatment options, either with RYGB or intensive medical weight loss utilizing a VLCD, while physicians assisted in selecting the most appropriate treatment option for each individual. This reflects real-world practice, where randomization may not be feasible. Second, the small sample size limits the possibility to draw definite conclusions and most participants were female, which may limit the ability to generalize the findings. Third, due to the non-randomized design, we could not exclude the potential differences in the self-motivation, self-efficacy and health literacy of the participants in the RYGB and VLCD groups. Lastly, our study had a short follow-up period. Despite these limitations, the findings are significant and highlight the possibility to use VLCD as an alternative treatment option for motivated patients with severe obesity who are unable to undergo bariatric surgery. Additional long-term follow-up studies are required to elucidate the mechanisms underlying differential weight loss and weight maintenance following VLCD and RYGB.

## 5. Conclusions

In summary, a 12-week VLCD was safe and effective and resulted in similar short-term weight loss and metabolic improvements compared with RYGB. The VLCD holds promise as an alternative treatment to MBS in motivated patients who are unable to undergo MBS. Otherwise, a VLCD can be used as an initial treatment of weight loss, followed by other strategies, including intensive behavioral therapy, diet counseling or even anti-obesity agents, to maintain weight loss as required. To prevent weight regain after food reintroduction, active follow-up programs including nutritional counseling, increased physical activity and behavior therapy are required to improve long-term, weight loss maintenance. Further large-scale studies with a longer follow-up period are needed to elucidate whether VLCD can be an alternative treatment to MBS.

## Figures and Tables

**Table 1:** Baseline characteristics, body composition and dietary intake of study participants.

Variables	RYGB (n = 16)	VLCD (n = 15)	*p*-Value
Sex, n (%)			0.252
Male	3 (18.8)	6 (40.0)	
Female	13 (81.3)	9 (60.0)	
Age, years	38.4 ± 9.3	31.8 ± 5.4	0.023
Weight, kg	109.2 ± 25.6	111.5 ± 23.3	0.795
Body mass index, kg/m^2^	41.3 ± 8.0	39.7 ± 7.0	0.576
Body fat mass, kg	54.5 ± 15.8	52.6 ± 14.5	0.736
% Body fat	49.4 ± 4.9	46.9 ± 5.0	0.163
Skeletal muscle mass, kg	30.2 ± 6.8	32.9 ± 6.6	0.280
% Skeletal muscle mass	27.9 ± 2.8	29.6 ± 3.0	0.102
Waist circumference, cm	115.2 ± 16.8	106.8 ± 9.5	0.311
Systolic blood pressure, mmHg	127.9 ± 16.4	127.0 ± 12.1	0.868
Diastolic blood pressure, mmHg	77.3 ± 7.6	83.7 ± 8.3	0.032
Pulse rate, bpm	87.4 ± 11.6	91.7 ± 11.4	0.302
Comorbidities			
Hypertension, n (%)	9 (56.3)	4 (26.7)	0.095
Dyslipidemia, n (%)	9 (56.3)	10 (66.7)	0.552
T2D, n (%)	7 (43.8)	5 (33.3)	0.552
Duration of T2D, year, median (IQR)	1 (0.5, 5.8)	0 (0, 0)	0.043
Medications			
Antidiabetic drug			
Insulin, n (%)	2 (12.5)	0 (0.0)	0.484
Oral hypoglycemic agent, n (%)	6 (37.5)	0 (0.0)	0.018
GLP-1 RA, n (%)	3 (18.8)	0 (0.0)	0.226
Antihypertensive drug			
ACEI, n (%)	3 (18.8)	0 (0.0)	0.226
Angiotensin receptor blocker, n (%)	2 (12.5)	1 (6.7)	1.000
Calcium channel blocker, n (%)	6 (37.5)	1 (6.7)	0.083
Beta blocker, n (%)	3 (18.8)	1 (6.7)	0.600
Diuretic, n (%)	1 (6.3)	0 (0.0)	1.000
Antihyperlipidemic drug			
Statin, n (%)	9 (56.3)	1 (6.7)	0.006
Ezetimibe, n (%)	1 (6.3)	0 (0.0)	1.000
Dietary intake			
Total energy intake, kcal/d	1693.8 ± 505.2	1789.8 ± 621.1	0.639
Protein intake, g/d	52.7 ± 19.9	90.3 ± 36.4	0.002
Fat intake, g/d	71.7 ± 24.8	71.2 ± 27.4	0.961
Carbohydrate intake, g/d	206.6 ± 75.3	195.2 ± 71.8	0.669

Data are presented as mean ± standard deviation and a number with percentage (%) or otherwise as indicated. RYGB, Roux-en-Y gastric bypass; VLCD, very low-calorie diet; T2D, type 2 diabetes; GLP-1 RA, glucagon-like peptide 1 receptor agonist; ACEI, angiotensin-converting enzyme inhibitor.

**Table 2:** Biochemical data of study participants at baseline.

Variables	RYGB (n = 16)	VLCD (n = 15)	*p*-Value
FPG, mg/dL			
All patients	123.7 ± 45.0	123.5 ± 59.0	0.994
Patients with T2D	142.3 ± 75.2	180.2 ± 75.2	0.362
Patients without T2D	109.2 ± 18.7	95.2 ± 14.9	0.086
HbA1c, %			
All patients	6.9 ± 1.8	7.0 ± 2.5	0.894
Patients with T2D	8.1 ± 2.3	9.5 ± 3.1	0.367
Patients without T2D	5.9 ± 0.4	5.7 ± 0.4	0.205
Total cholesterol, mg/dL	178.3 ± 33.1	216.8 ± 38.9	0.006
Triglyceride, mg/dL	144.2 ± 66.2	133.5 ± 68.7	0.661
HDL cholesterol, mg/dL	46.4 ± 7.8	46.7 ± 10.5	0.930
LDL cholesterol, mg/dL	131.9 ± 38.4	158.6 ± 38.5	0.063
Uric acid, mg/dL	6.5 ± 1.9	7.2 ± 1.8	0.257
Serum creatinine, mg/dL	0.7 ± 0.3	0.7 ± 0.1	0.476
eGFR, mL/min/1.73 m^2^	107.7 ± 25.4	118.7 ± 14.3	0.148
AST, U/L	33.8 ± 19.9	50.9 ± 62.0	0.319
ALT, U/L	36.9 ± 23.3	77.9 ± 113.5	0.182

Data are presented as mean ± standard deviation. RYGB, Roux-en-Y gastric bypass; VLCD, very low-calorie diet; T2D, type 2 diabetes; FPG, fasting plasma glucose; HbA1c, hemoglobin A1c; HDL, high-density lipoprotein; LDL, low-density lipoprotein; eGFR, estimated glomerular filtration rate; AST, aspartate aminotransferase; ALT, alanine transaminase.

**Table 3:** Body composition and dietary intake of study participants at 4 weeks and 12 weeks after RYGB or VLCD.

Variables	RYGB (n = 16)	VLCD (n = 15)	Mean Difference (95% CI)	*p*-Value
Weight, kg				
4 weeks	100.8 ± 0.8	105.5 ± 0.8	−4.7 (−6.8, −2.7)	<0.001
12 weeks	91.4 ± 0.8	96.1 ± 0.8	−4.7 (−6.8, −2.7)	<0.001
Body mass index, kg/m^2^				
4 weeks	38.0 ± 0.3	37.6 ± 0.3	0.4 (−0.4, 1.1)	0.345
12 weeks	34.6 ± 0.3	34.2 ± 0.3	0.4 (−0.4, 1.1)	0.345
Body fat mass, kg				
4 weeks	49.8 ± 0.9	49.2 ± 1	0.6 (−1.8, 3)	0.619
12 weeks	40.6 ± 0.9	40.0 ± 1	0.6 (−1.8, 3)	0.619
% Body fat				
4 weeks	48.8 ± 0.8	46.3 ± 0.8	2.5 (0.5, 4.5)	0.014
12 weeks	43.6 ± 0.8	41.1 ± 0.8	2.5 (0.5, 4.5)	0.014
Skeletal muscle mass, kg				
4 weeks	28.1 ± 0.3	31.3 ± 0.3	−3.3 (−4.1, −2.4)	<0.001
12 weeks	27.5 ± 0.3	30.8 ± 0.3	−3.3 (−4.1, −2.4)	<0.001
% Skeletal muscle mass				
4 weeks	28.1 ± 0.4	29.8 ± 0.4	−1.7 (−2.7, −0.8)	<0.001
12 weeks	30.5 ± 0.4	32.2 ± 0.4	−1.7 (−2.7, −0.8)	<0.001
Waist circumference, cm				
4 weeks	111.3 ± 1.8	101.1 ± 1.8	10.3 (5.2, 15.3)	<0.001
12 weeks	97.0 ± 1.8	94.7 ± 1.8	2.3 (−2.8, 7.4)	0.369
Dietary intake				
Total energy intake, kcal/d				
4 weeks	304.7 ± 38.1	824.8 ± 39.4	−520.1 (−627.6, −412.6)	<0.001
12 weeks	679.3 ± 38.1	875 ± 39.4	−195.7 (−303.2, −88.3)	<0.001
Protein intake, g/d				
4 weeks	20.6 ± 4.6	96.3 ± 4.7	−75.8 (−86.4, −65.2)	<0.001
12 weeks	43.8 ± 4.6	119.6 ± 4.7	−75.8 (−86.4, −65.2)	<0.001
Fat intake, g/d				
4 weeks	14.6 ± 2.7	23.1 ± 2.8	−8.5 (−16.1, −1.0)	0.027
12 weeks	31.7 ± 2.7	28.7 ± 2.8	3.0 (−4.6, 10.6)	0.438
Carbohydrate intake, g/d				
4 weeks	23.7 ± 4.7	48.8 ± 4.8	−25.2 (−38.3, −12)	<0.001
12 weeks	44.7 ± 4.7	48.9 ± 4.8	−4.2 (−17.4, 8.9)	0.528

Data are presented as mean ± standard error or mean difference (95% confidence intervals). RYGB, Roux-en-Y gastric bypass; VLCD, very low-calorie diet.

**Table 4:** Changes in biochemical data of study participants at 12 weeks after RYGB and VLCD.

Variables	RYGB (n = 16)	VLCD (n = 15)	Median Difference (95% CI)	*p*-Value
FPG, mg/dL				
All patients	−16.5 (−30.0, −6.0)	−10.0 (−29.0, −3.0)	−12.0 (−53.3, 29.3)	0.557
Patients with T2D	−22.0 (−46.0, 5.0)	−118.0 (−136.0, −11.0)	96 (−58.2, 250.2)	0.195
Patients without T2D	−9.0 (−28.0, −6.0)	−5.5 (−11.0, −2.0)	−2 (−18.6, 14.6)	0.802
HbA1c, %				
All patients	−0.5 (−0.9, −0.3)	−0.4 (−1.0, −0.0)	−0.2 (−1.6, 1.2)	0.766
Patients with T2D	−0.6 (−3.4, 0.0)	−4.1 (−6.0, −1.0)	3.6 (−0.8, 7.9)	0.100
Patients without T2D	−0.5 (−0.7, −0.3)	−0.2 (−0.4, 0.0)	−0.2 (−0.6, 0.1)	0.160
Total cholesterol, mg/dL	−16.5 (−35.0, 5.5)	−24.0 (−44.0, 2.0)	5 (−24.1, 34.1)	0.727
Triglyceride, mg/dL	−38.0 (−69.5, −12.0)	−22.0 (−54.0, −3.0)	−28 (−64.5, 8.5)	0.128
HDL cholesterol, mg/dL	−3.0 (−10.0, 7.5)	−5.0 (−13.0, 1.0)	3 (−7.1, 13.1)	0.550
LDL cholesterol, mg/dL	−25.0 (−34.0, −9.0)	−28.0 (−35.0, 8.0)	−1 (−29.5, 27.5)	0.943
Uric acid, mg/dL	−0.7 (−1.5, 0.3)	0.2 (−1.4, 0.9)	−0.9 (−2.5, 0.7)	0.255
Serum creatinine, mg/dL	0.0 (−0.1, 0.1)	0.0 (−0.1, 0.0)	0 (−0.1, 0.1)	0.776
eGFR, mL/min/1.73 m^2^	−0.2 (−4.6, 2.6)	0.0 (−2.6, 3.0)	−0.3 (−5.6, 5)	0.908
AST, U/L	4.0 (−2.0, 14.0)	−5.0 (−21.0, 0.0)	9 (−6.3, 24.3)	0.237
ALT, U/L	2.0 (−12.0, 9.0)	−17.0 (−31.0, −2.0)	19 (1.1, 36.9)	0.039

Data are presented as median (interquartile range) or mean difference (95% confidence interval). RYGB, Roux-en-Y gastric bypass; VLCD, very low-calorie diet; T2D, type 2 diabetes; FPG, fasting plasma glucose; HbA1c, hemoglobin A1c; HDL, high-density lipoprotein; LDL, low-density lipoprotein; eGFR, estimated glomerular filtration rate; AST, aspartate aminotransferase; ALT, alanine transaminase.

## Data Availability

The data presented in the current study are not publicly available owing to privacy and ethical restrictions. However, data are available from the corresponding author upon reasonable request.
